# Plasma Quantitative Lipid Profiles: Identification of CarnitineC18:1-OH, CarnitineC18:2-OH and FFA (20:1) as Novel Biomarkers for Pre-warning and Prognosis in Acute Myocardial Infarction

**DOI:** 10.3389/fcvm.2022.848840

**Published:** 2022-04-11

**Authors:** Jun Liu, Liangqiu Tang, Qiqi Lu, Yi Yu, Qiu-Gui Xu, Shanqiang Zhang, Yun-Xian Chen, Wen-Jie Dai, Ji-Cheng Li

**Affiliations:** ^1^Medical Research Center and Department of Cardiology, Yue Bei People's Hospital, Shantou University Medical College, Shaoguan, China; ^2^The Central Laboratory, Yangjiang People's Hospital, Yangjiang, China; ^3^Department of Histology and Embryology, Shaoguan University School of Medicine, Shaoguan, China; ^4^Institute of Cell Biology, Zhejiang University, Hangzhou, China

**Keywords:** lipid metabolites, UPLC-MS/MS, machine learning, quantitative lipid profile, AMI

## Abstract

This study was aimed to determine the association between potential plasma lipid biomarkers and early screening and prognosis of Acute myocardial infarction (AMI). In the present study, a total of 795 differentially expressed lipid metabolites were detected based on ultra-performance liquid chromatography tandem mass spectrometry (UPLC-MS/MS). Out of these metabolites, 25 lipid metabolites were identified which showed specifical expression in the AMI group compared with the healthy control (HC) group and unstable angina (UA) group. Then, we applied the least absolute shrinkage and selection operator (LASSO) and support vector machine-recursive feature elimination (SVM-RFE) methods to obtain three lipid molecules, including CarnitineC18:1-OH, CarnitineC18:2-OH and FFA (20:1). The three lipid metabolites and the diagnostic model exhibited well predictive ability in discriminating between AMI patients and UA patients in both the discovery and validation sets with an area under the curve (AUC) of 0.9. Univariate and multivariate logistic regression analyses indicated that the three lipid metabolites may serve as potential biomarkers for diagnosing AMI. A subsequent 1-year follow-up analysis indicated that the three lipid biomarkers also had prominent performance in predicting re-admission of patients with AMI due to cardiovascular events. In summary, we used quantitative lipid technology to delineate the characteristics of lipid metabolism in patients with AMI, and identified potential early diagnosis biomarkers of AMI via machine learning approach.

## Introduction

Acute myocardial infarction (AMI) continues to be associated with high morbidity and mortality globally (1). According to the World Health Organization (WHO) estimation, the number of deaths due to AMI accounted for 31% of the global deaths (2). According to the WHO report, in 2020, ischemic heart disease ranked first among the causes of death worldwide. In 2019, the number of new deaths was over 2 million, and the total number of deaths was estimated to reach 8.9 million (https://www.who.int/). In China, the mortality rate of AMI also showed a rapidly increasing trend with nearly 2.5 million AMI related deaths each year (3). Depending on incomplete statistics, ~10% of emergency department patients with chest pain are diagnosed with AMI (4). Although the onset of AMI is generally insidious, the disease can progress rapidly and may cause cardiac arrest (5). AMI can also cause serious complications, including heart failure and malignant arrhythmia. Early diagnosis and pre-warning of AMI are essential for timely preventing the disease process, which can be of great significance to reduce the mortality and improve the prognosis of AMI.

Clinically, the diagnosis of AMI is mainly relied on the presence of chest pain and an abnormal electrocardiogram, while patients with unstable angina (UA) may present with similar clinical manifestations (6). Therefore, distinguishing between AMI and UA is challenging due to the lack of atypical clinical presentation. In recent decades, the biomarkers of myocardial injury have been used in the diagnosis of AMI. Karmen et al. first reported the significant increase in the Aspartate aminotransferase (AST) levels in AMI patients, and since then, AST has been used as a biomarker for clinical diagnosis of AMI (7). However, with more research conducted, increasing evidence demonstrated that AST lacked specificity for diagnosing myocardial injury, and the clinical application of AST as a biomarker for AMI was not that optimistic (8). In subsequent decades, creatine kinase (CK) and lactate dehydrogenase (LDH) have been widely used in the early diagnosis of AMI (9). However, their specificity remained problematic as an early biomarker, especially in patients suffering from muscle injury and liver diseases (10). Cardiac troponin levels have been demonstrated to be more specific and sensitive for AMI than CK-MB and LDH (11). However, troponin levels can only be detected within 4–6 h after myocardial injury, and its 100% sensitivity time is 8–12 h after AMI. This may lead to serious injury of the myocardial tissue in patients with AMI, and may cause death or serious complications. Therefore, the identification of potential biomarkers for the early diagnosis of AMI with high sensitivity and specificity is very important.

Atherosclerosis is the major risk factor that can lead to AMI (12). As it progresses, the atherosclerotic plaque can rupture causing the vascular lumen obstruction, which in turn can lead to myocardial ischemic injury and AMI. Presently, with the rapid development and application of multi-omics, increasing researchers seek to identify potential novel biomarkers and explore disease pathogenesis via omics technology (13–15). The role of transcriptome, proteomics and metabolomics have been described previously in screening studies of various diseases (16–18). Metabolomics can provide an integrated landscape of systemic metabolic disorder, and altered metabolic process and pathways could better reflect the disease process (19). The metabolites are also more stable than RNA and proteins in serum, which makes them ideal biomarkers. A number of studies have already suggested that lipid metabolites are closely related to the occurrence of AMI (20, 21). However, the relationship between lipid metabolism and AMI needs to be further clarified.

Lipidomic is a subfield of metabolomics, which can detect a variety of lipid molecules (22). It has been shown that the disorder of lipid metabolism is associated with vascular inflammatory response and oxidative stress. Vascular inflammatory response and oxidative stress are important risk factors for the formation of atherosclerosis and AMI. Marwa et al. found that lipid metabolites can be used as potential biomarkers for the early diagnosis of ataxia syndrome (23). Mei et al. reported that lipid metabolites may serve as potential biomarkers for the early diagnosis of Parkinson's disease (24). Recently, Ju Yeon et al. identified differentially expressed lipid metabolic molecules in patients with AMI and UA by ultra-high performance liquid chromatography/quadrupole time of flight mass spectrometry, which suggested the promising potential of lipid metabolites in AMI diagnosis (25). However, the characteristics of lipid metabolism in AMI have not been fully elucidated, and the diagnostic value of lipid biomarkers remains to be explored. In this study, ultra-high performance liquid chromatography-tandem mass spectrometry (UPLC-MS/MS) was performed to characterize the lipid metabolism profile in AMI based on the absolute quantitative lipid technology. Furthermore, the differentially expressed lipid molecules and enriched metabolic pathways in AMI patients, HC subjects and UA patients were identified in order to elucidate the potential role of lipid molecules in detecting the occurrence of AMI.

## Materials and Methods

### Collection and Grouping of Samples

The design and procedure of the present study were in accordance with the Helsinki Declaration and approved by the ethics committee of the Yue Bei People's Hospital Affiliated to Shantou University Medical College (China, KY-2021-050). Informed consent was obtained from all patients and healthy volunteers prior to study. The enrolled subjects were split into the discovery set and the validation set. There were 90 subjects in the discovery cohort (30 HC subjects, 30 UA patients and 30 AMI patients) and 60 patients in the validation cohort (30 UA patients and 30 AMI patients). A total of 150 patients were enrolled in this study, and basic clinical information of all patients was acquired (Table 1). Subjects with severe hypertension, pulmonary dysfunction, arrhythmia, liver and kidney dysfunction, tumor diseases, mental diseases, pregnant and lactating women, as well as infectious and congenital diseases were excluded.

**Table 1:** The clinical information of patients.

		**Discovery Set**			**Validation Set**	
**Characteristic**		**HC**	**UA1**	**AMI1**	**UA2**	**AMI2**
Sex	Male	21	21	23	21	23
	Female	9	9	7	9	7
Age	< =60	14	13	14	13	15
	>60	16	17	16	17	15
CKMB			15.35	54.45	13.75	37.5
			(13.43, 18.48)	(23.77, 194.47)	(11.03, 17.88)	(24.6, 160.8)
TC			4.88 ± 0.86	4.85 ± 1.05	4.57 ± 1.06	5.13 ± 1.25
TG			1.77 ± 1.31	1.34 ± 0.84	1.48 ± 0.66	1.57 ± 1.14
HDL			1.23 ± 0.29	1.27 ± 0.73	1.15 ± 0.21	1.13 ± 0.23
LDL			2.88 ± 0.79	2.94 ± 0.9	2.65 ± 0.81	3.1 ± 1.04

### Extraction and Analysis of Lipid Metabolites

Plasma samples were taken out from the−80°C freezer and thawed at room temperature. After post-thawing, all samples were centrifuged and vortexed for 10 s to ensure uniform mixing, then these samples were centrifuged at 3,000 g for 5 min at 4°C. After centrifugation, the serum was completely removed and transferred to a clean Eppendorf tube. Subsequently, 1 ml of lipid extraction solution and internal standards mixture were added to the Eppendorf tube and mixed by vortex for 2 min (26, 27). The mixed samples were emulsified by sonication for 5 min, mixed with 500 uL water, vortexed for 1 min, and then centrifuged at 4°C 12,000 g for 10 min. The 500 uLsupernatant was collected and dried with nitrogen and re-dissolved with 100 uL of mobile phase B. After vortex oscillation for 1 min, the sample was centrifuged at 14,000 g for 15 min at 4°C, and UPLC-MS / MS analysis was carried out.

### Liquid Chromatography and Mass Spectrometry

Ultra Performance Liquid Chromatography (UPLC, ExionLC™ AD, https://sciex.com.cn/) and Tandem Mass Spectrometry (MS/MS, QTRAP® 6500+, https://sciex.com.cn/) are the main instrument system for data acquisition. The chromatographic columns from Thermo Accucore™C30 (2.6 μm, 2.1 mm × 100 mm i.d.) were used. The solvent system was as follows: A, acetonitrile/water (60/40, V/V, 0.1% formic acid, 10 mmol/L ammonium formate); B, acetonitrile/isopropanol (10/90, VV/V, 0.1% formic acid, 10 mmol/L ammonium formate). Gradient program was *t* = 0 min: A/B (80:20, V/V); *t* = 2.0 min: A/B (70:30, V/V); *t* = 4 min: A/B (40:60, V/V); *t* = 9 min: A/B (15:85, V/V), *t* = 14 min: A/B (10:90, V/V); *t* = 15.5 min: A/B (5:95, V/V); *t* = 17.3 min: A/B (5:95, V/V); *t* = 17.5 min: A/B (80:20, V/V); *t* = 20 min: A/B (80:20, V/V); and flow rate was 0.35 ml/min, with temperature was 45°C. Subsequently, the effluent was alternatively connected to an electrospray ionization (ESI) -triple quadrupole-linear ion trap (QTRAP)-MS. Linear ion trap and triple quadrupole (QQQ) scans were acquired on a triple quadrupole-linear ion trap mass spectrometer (QTRAP), QTRAP® 6500+ LC-MS/MS System, equipped with an ESI Turbo Ion-Spray interface, operating in positive and negative ion mode and controlled by Analyst 1.6.3 software (Sciex). Lipids contents were detected by MetWare (http://www.metware.cn/) based on the AB Sciex QTRAP 6500 LC-MS/MS platform.

### Qualitative and Quantitative Analysis

The MWDB (metware database) was constructed based on the standard materials to qualitatively analyze the data detected by mass spectrometry. The multiple reaction monitoring (MRM) mode of triple quadrupole mass spectrometry was applied for the quantification of analytes.

### Bioinformatics and Statistical Analysis

The R programming language (version 4.1.1) was applied for statistical analyses and generation of graph. The characteristic ions of each lipid metabolites were processed by the multiple reaction monitoring based on the Metware database, and then MultiQuant software was performed to analyze the chromatogram review and peak area integration of the off-board mass spectrometry file of the sample. Each chromate graphic peak represents a lipid metabolite, and the area under the peak corresponds to the relative content. The qualitative and quantitative analysis results of all lipid metabolites were calculated based on the linear equation and calculation formula. The variable importance in projection (VIP) was calculated based on the orthogonal partial least squares discriminant analysis (OPLS-DA), and the *P*-value and fold change (FC) of the nonparametric test were used in combination to screen the differential lipid metabolites. Then, the cut offs of VIP ≥ 1, FC > 1.5 or FC <0.66 and *P* < 0.05 were used as standards to screen differential lipid metabolites. Kyoto Encyclopedia of genes and genes (KEGG) was performed to annotate the function of the enriched differential lipid metabolites. The least absolute shrinkage and selection operator (LASSO) regression and support vector machine (SVM) methods were used to further select features, and logistic regression was performed to construct a diagnostic model. LASSO regression compresses the coefficients of redundant or uninformative variables to zero based on constructing a penalty function, with the feature with non-zero coefficients are finally filtered out (28). LAASO regression was characterized by fitting generalized linear models with both variable selection and complexity adjustment. SVM is a classical binary classification method, which is widely applied in medical diagnosis (29). The basic principle of SVM learning is to solve the separation hyperplane which can correctly divide the training data set and has the largest geometric interval. Subsequently, the diagnostic power of the model was evaluated by the receiver operating characteristic (ROC) analysis. Finally, univariate and multivariate logistic regression analyses were applied to assess the risk factors for AMI.

## Results

### Identification and Multivariate Statistical Analysis of Lipid Metabolites

**Figure 1:**
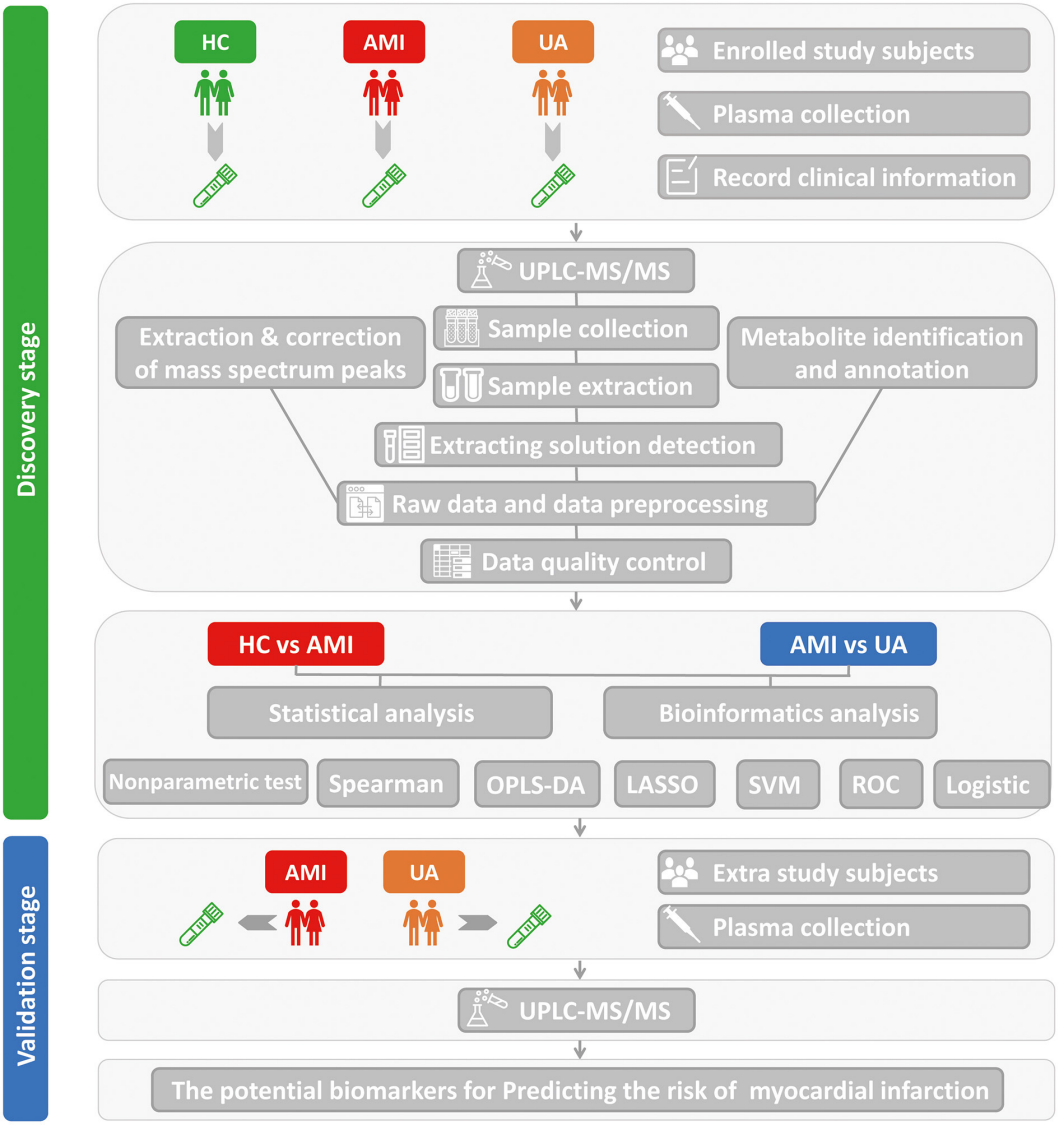
The workflow of this study.

**Figure 2:**
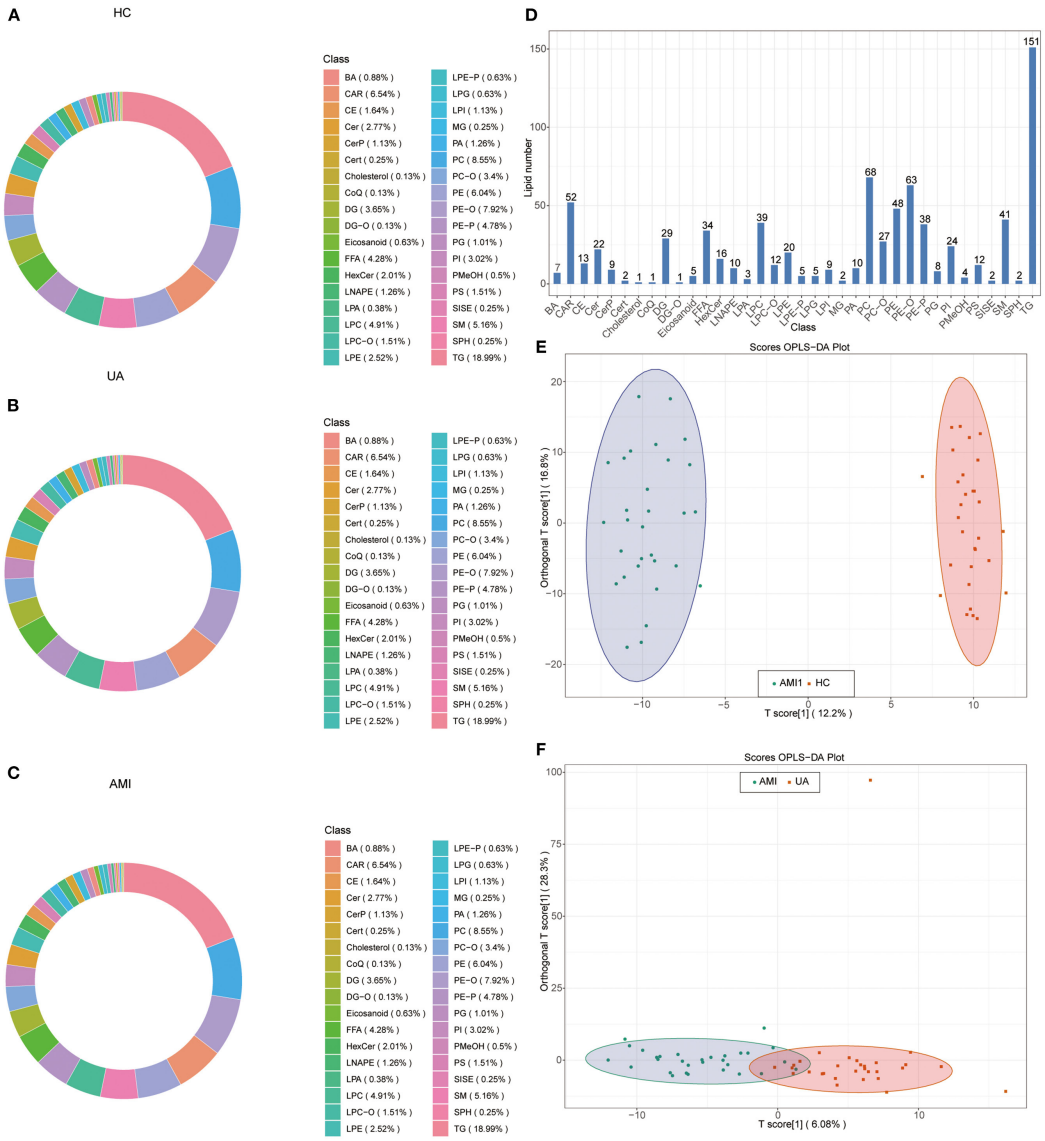
Identification of lipid components and orthogonal partial least squares discriminant analysis (OPLS-DA). Circular diagram of lipid subclass composition in healthy control (HC) group **(A)**, unstable angina (UA) group **(B)**, and acute myocardial infarction (AMI) group **(C,D)**. Total detected lipid subclasses and the number of lipid compounds contained in each subclass. OPLS-DA score plot in AMI vs. HC **(E)** and AMI vs. UA **(F)**.

The workflow diagram demonstrated the study design process, including detailed grouping information, experimental flow, and identification of lipid biomarkers of AMI (Figure 1). A total of 795 differential lipid metabolites were detected using quantitative lipid detection based on an UPLC-MS/MS. In total, 36 differential lipid subclasses were detected in three groups (Figures 2A–C). Of 36 lipid subclasses, the most abundant was TG (151 lipid molecules), followed by phosphatidylcholine (PC) and phosphatidylethanolamine-ethers (PE-O) with 68 and 63 lipid molecules, respectively (Figure 2D). The orthogonal partial least squares discriminant analysis (OPLS-DA) revealed significantly differential lipid metabolites between the AMI group and the HC and UA groups (Figures 2E,F).

### Identification and Enrichment Analysis of Differential Lipid Metabolites

**Figure 3:**
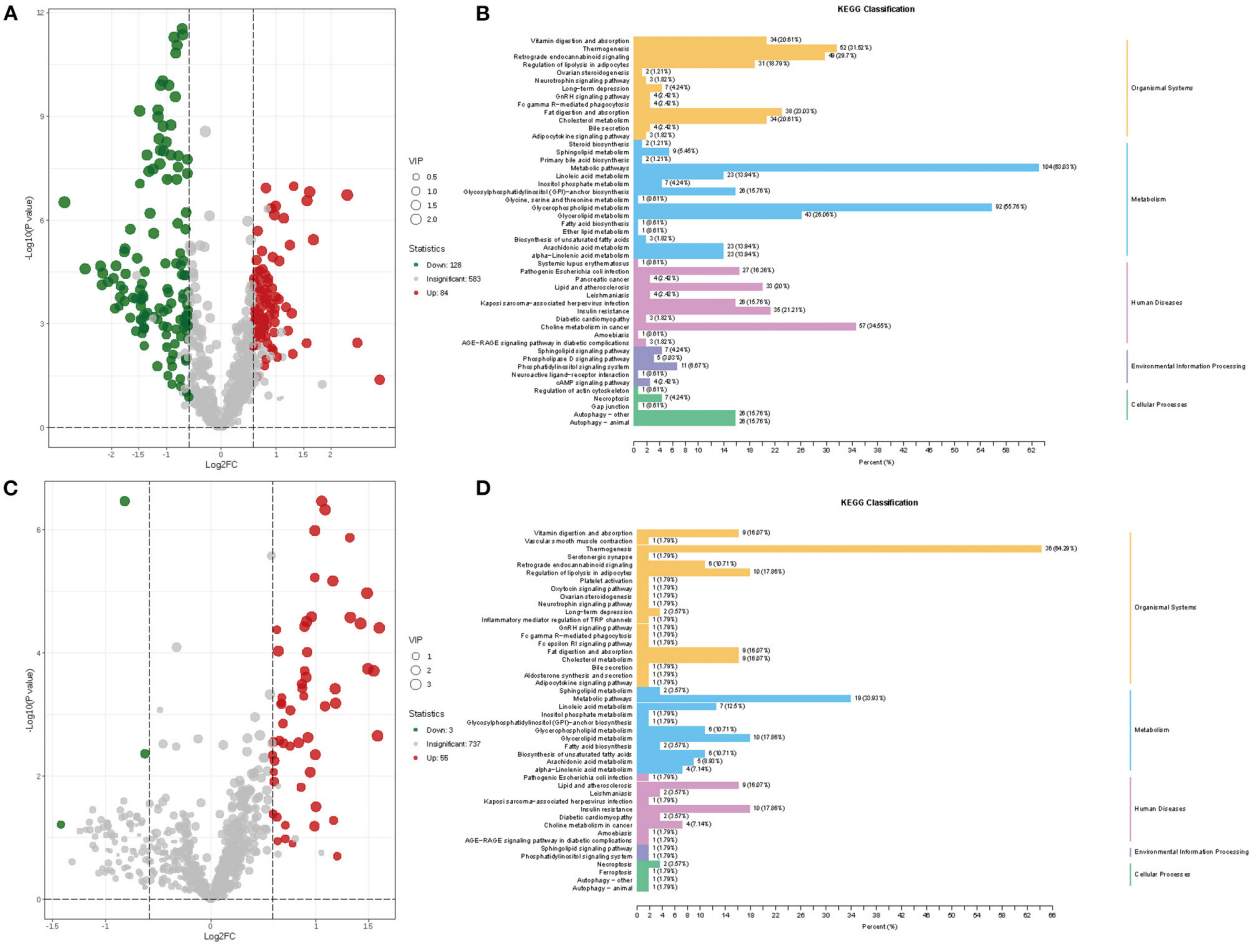
Screening and enrichment analysis of differential lipid metabolites. **(A)** Volcano plot showing differential lipid metabolites between AMI and HC. **(B)** KEGG enrichment analysis for differential lipid metabolites between AMI and HC. **(C)** Volcano plot showing differential lipid metabolites between AMI and UA. **(D)** KEGG enrichment analysis for differential lipid metabolites between AMI and UA.

**Figure 4:**
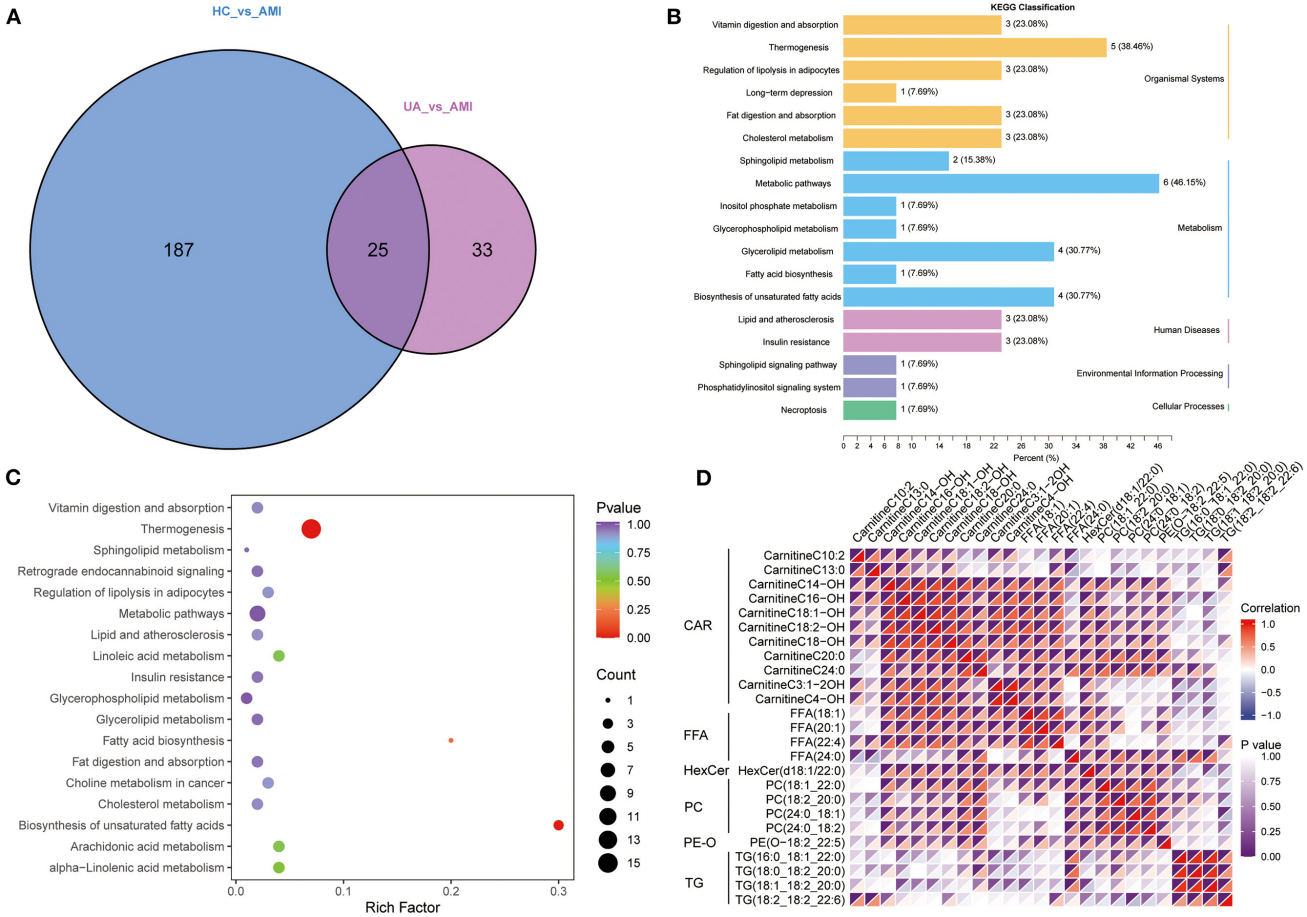
Acquisition of common differential lipid metabolites. **(A)**. Venn diagram shows the common differential lipid metabolites between the group. **(B)**. The annotation results are classified according to the type of KEGG pathway. **(C)**. KEGG pathway enrichment analysis for common differential lipid metabolites. **(D)**. A cluster analysis of common differential lipid metabolites.

A total of 212 differential lipid metabolites were identified between the AMI group and the HC group based on VIP value >1, *P* < 0.05, and FC >1.5 or FC < 0.6. Of these, 128 lipid metabolites were downregulated, while 84 lipid metabolites were upregulated in the AMI group (Figure 3A). Subsequently, KEGG enrichment analysis was performed to explore the potential function of these differential lipid molecules, and the results suggested that these differential lipid metabolites were mainly associated with the regulation of lipolysis in adipocytes, fat digestion and absorption, metabolic pathways, and glycerophospholipid metabolism (Figure 3B). In addition, we also screened differential lipid metabolites between the AMI group and the UA group. The results showed that 55 lipid metabolites were upregulated, while 3 lipid metabolites were downregulated in the AMI group (Figure 3C). KEGG analysis revealed that these differential metabolites were mainly enriched in thermogenesis, metabolic pathways, and glycerol lipid metabolism (Figure 3D). Furthermore, Venn diagram showed the overlapping of differential lipid metabolites for two comparisons (AMI vs. HC and AMI vs. UA), and 25differential lipid metabolites were found to be specifically expressed in the AMI group (Figure 4A). KEGG pathway analysis suggested that these 25 specific differential lipid metabolites were mainly enriched in thermogenesis, metabolic pathways, glycerol lipid metabolism and fatty add biosynthesis (Figures 4B,C). Co-expression cluster analysis revealed that these 25 differential lipid molecules were mainly classified as acyl carnitine (CAR), free fatty acid (FFA), hexosylceramides (HexCer), PC, PE-O, and TG (Figure 4D). Further, there was a strong correlation between CAR and FFA, and PC was strongly associated with TG.

### Construction of a Diagnostic Model for AMI

**Figure 5:**
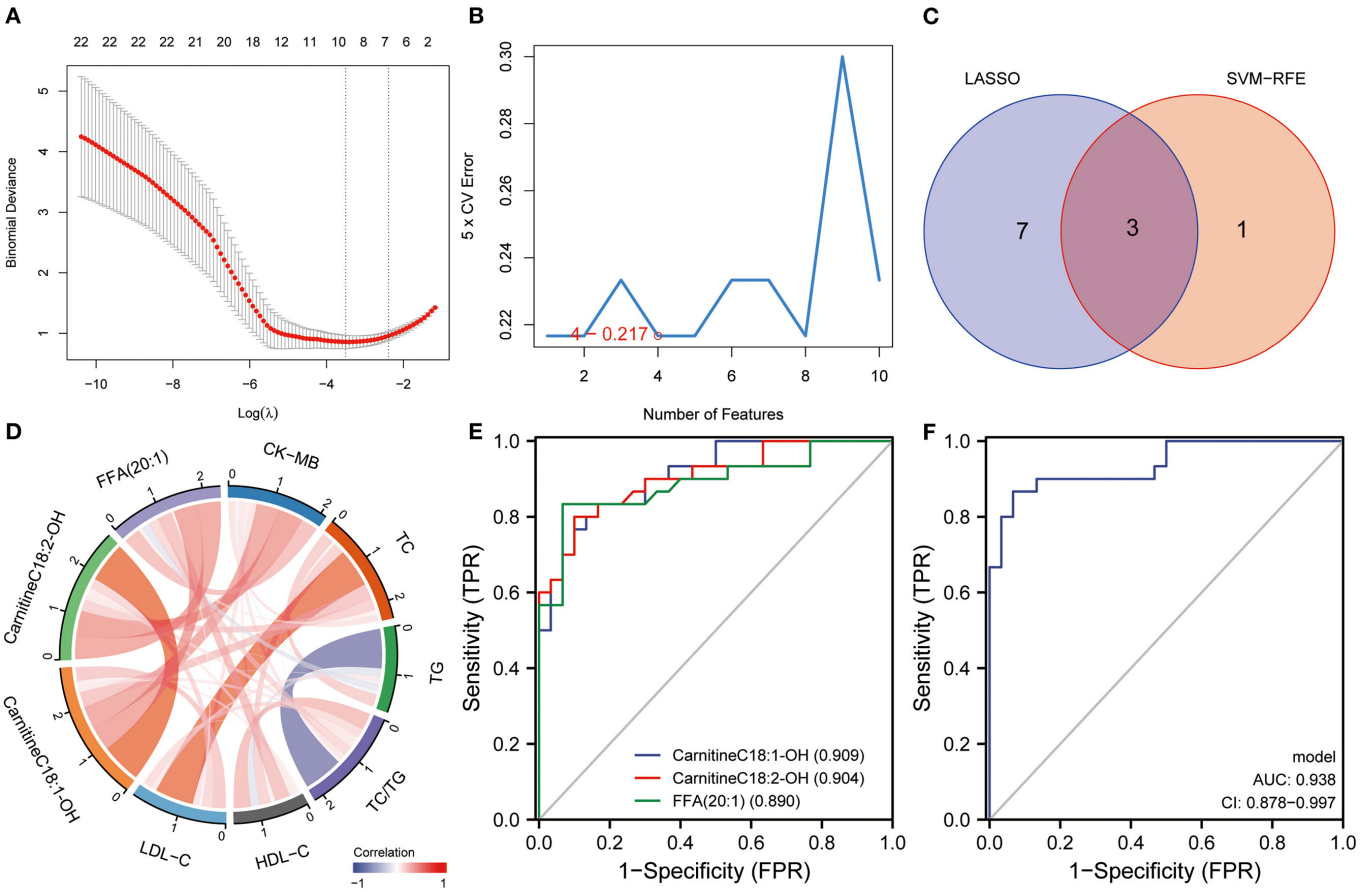
Identification of lipid diagnostic biomarkers. **(A)** LASSO and **(B)** SVM-RFE algorithms in the discovery set. **(C)** Veen diagram shows the lipid biomarkers selected by the LASSO and SVM-RFE algorithms. **(D)** Correlation analysis between lipid biomarkers and routine clinical indexes. **(E)** The diagnostic performance of lipid biomarkers in distinguishing AMI from UA was evaluated by ROC analysis in discovery set. **(F)** The diagnostic performance of diagnostic model in distinguishing AMI from UA was evaluated by ROC analysis in discovery set.

**Figure 6:**
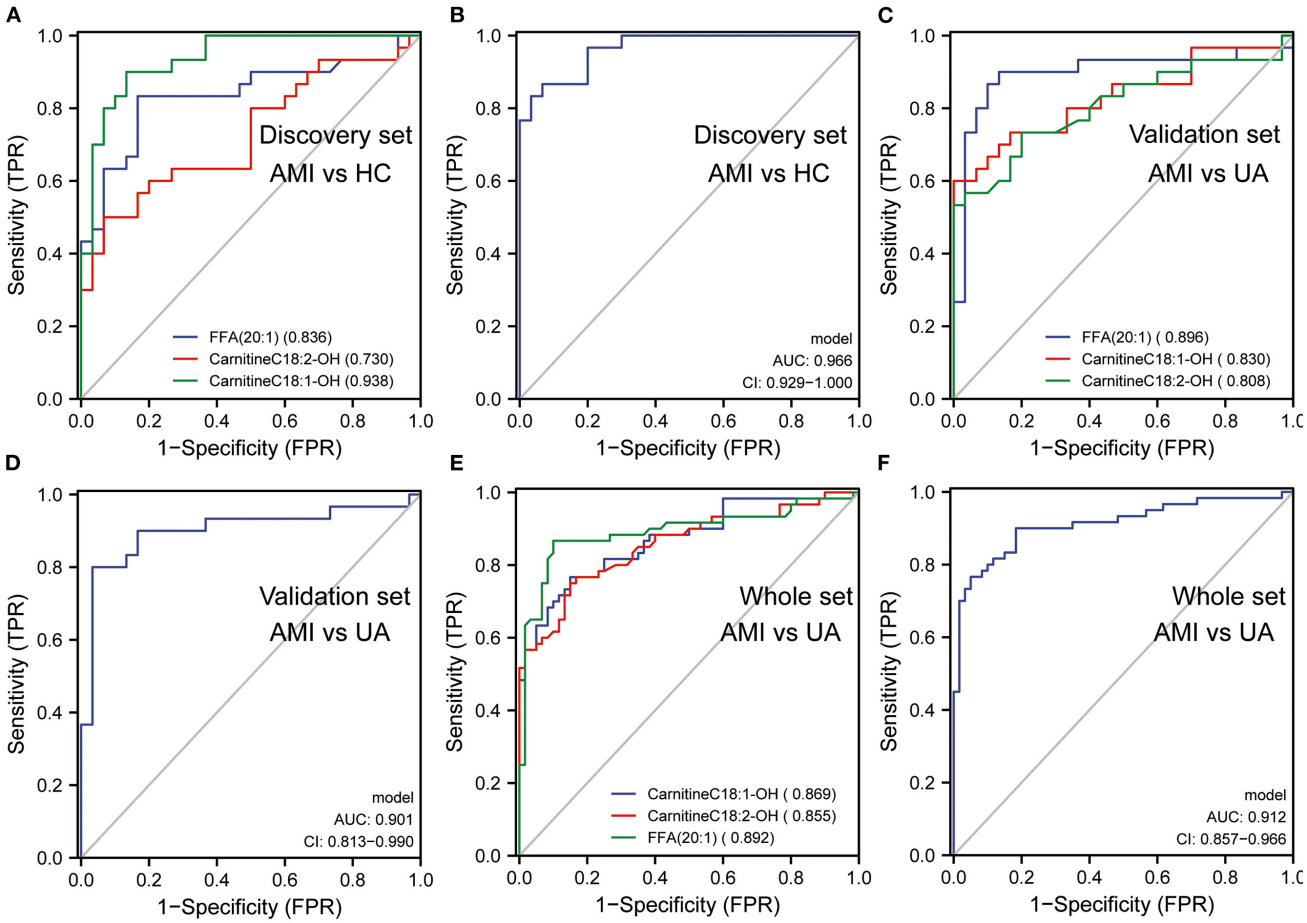
The diagnostic efficacy of lipid biomarkers in AMI. **(A)** The diagnostic performance of lipid biomarkers in distinguishing AMI from HC was evaluated by ROC analysis in discovery set. **(B)** The diagnostic performance of diagnostic model in distinguishing AMI from HC was evaluated by ROC analysis in discovery set. **(C)** The diagnostic performance of lipid biomarkers in distinguishing AMI from UA was evaluated by ROC analysis in validation set. **(D)** The diagnostic performance of diagnostic model in distinguishing AMI from UA was evaluated by ROC analysis in validation set. **(E)** The diagnostic performance of lipid biomarkers in distinguishing AMI from UA was evaluated by ROC analysis in whole set. **(F)** The diagnostic performance of diagnostic model in distinguishing AMI from UA was evaluated by ROC analysis in whole set.

The LASSO regression analysis was used to screen the lipid biomarkers in the discovery set, which could distinguish AMI patients from UA patients. And then, 10 lipid biomarkers with unique expression in the AMI group were identified (Figure 5A). SVM-RFE algorithm was applied to select the optimum feature, and four lipid metabolites were identified as potential biomarkers for AMI (Figure 5B). In order to filter out relatively more robust biomarkers, we took the intersection of the two algorithms and obtained three lipid biomarkers, including FFA (20:1), CarnitineC18:2-OH and CarnitineC18:1-OH (Figure 5C). The results of ROC curve analysis showed that the diagnostic performance of FFA (20:1), CarnitineC18:2-OH, and CarnitineC18:1-OH had good performance in distinguishing AMI patients from UA patients, and the AUC of these three lipid biomarkers was 0.89, 0.904 and 0.909, respectively (Figure 5E). Furthermore, logistic regression was applied to construct a diagnostic model based on the three lipid biomarkers in the discovery set. The diagnostic model was able to distinguish well between AMI patients and UA patients with AUC 0.938 (Figure 5F). Then, the ROC analysis was used to determine the performance of the three lipid biomarkers in distinguishing between HC subjects and AMI patients. The results showed that FFA (20:1) and CarnitineC18:1-OH had better performance in distinguishing HC subjects from AMI patients, and the AUC were 0.836 and 0.938, respectively (Figure 6A). We combined these three lipid biomarkers to construct a diagnostic model by logistic regression, which showed good efficacy in distinguishing between AMI patients and HC subjects, with an AUC of 0.966 (Figure 6B). In the validation set, the three lipid metabolites and diagnostic model also presented a relatively good performance in distinguishing between AMI patients and UA patients, and the AUC was over 0.8 (Figures 6C,D). We integrated the discovery set and the verification set into a whole set. ROC analysis showed that the three lipid biomarkers presented excellent diagnostic ability in distinguishing between AMI patients and UA patients in the whole set, with an AUC > 0.85 (Figure 6E). As expected, the diagnostic model combining FFA (20:1), CarnitineC18:2-OH and CarnitineC18:1-OH performed well on the whole set with an AUC of 0.912 (Figure 6F). Collectively, these results indicated that the three differential lipid metabolites had excellent promise in discriminating AMI patients from UA patients and HC subjects, and may serve as potential risk biomarkers for AMI.

### Correlation Between Lipid Biomarkers and Clinical Indexes

**Figure 7:**
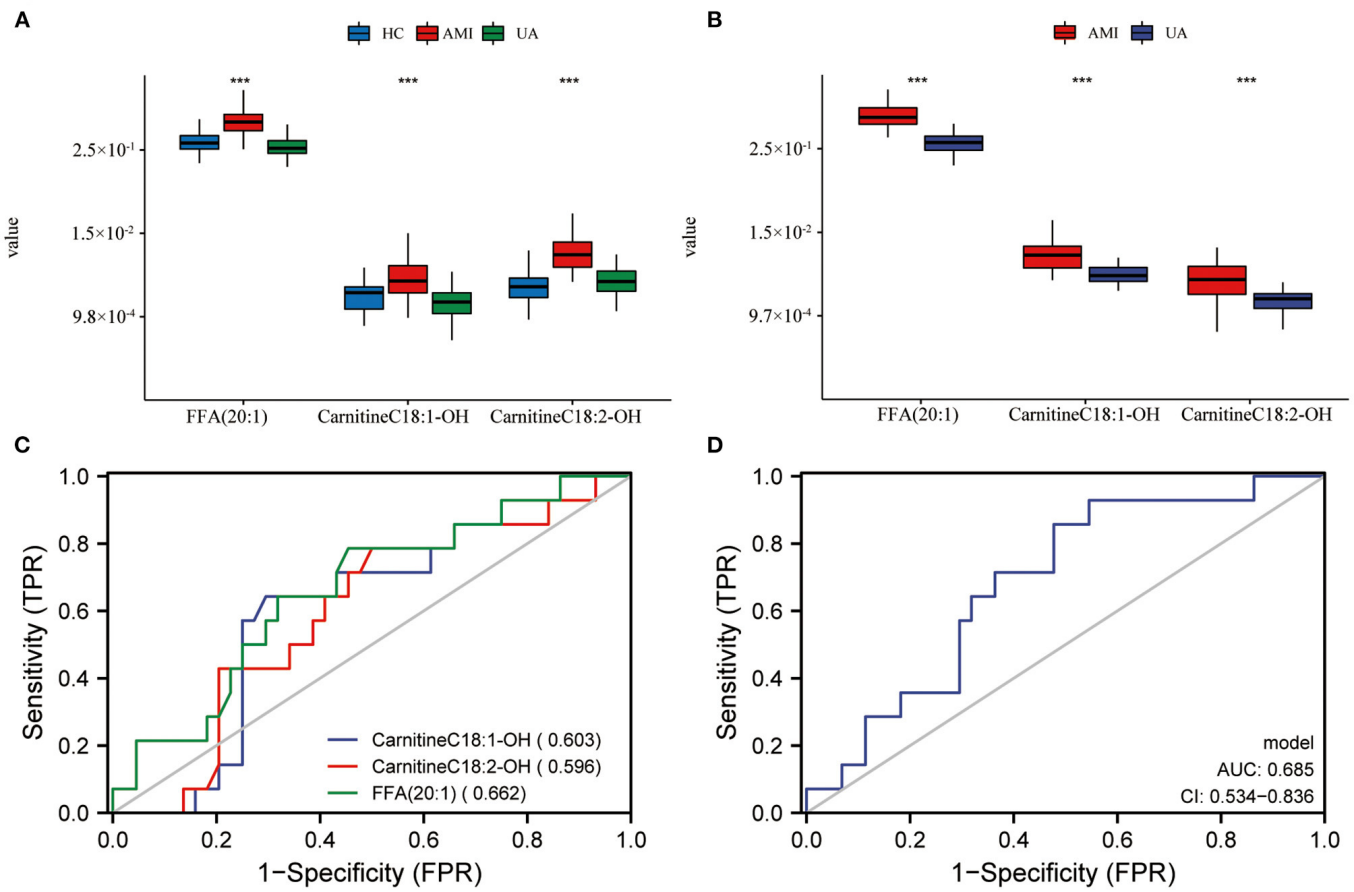
Prognostic evaluation of lipid biomarkers and expression in AMI patients. Expression level of lipid biomarkers in discovery set **(A)** and validation set **(B)**. The prognostic evaluation of lipid biomarkers **(C)** and model **(D)** in whole set. ***P < 0.001.

Currently, CK-MB is the most commonly used index in clinical diagnosis of AMI, while, TC, TG, HDL-C and LDL-C are also common parameters related to AMI in the clinic. In this study, we evaluated the serum levels of CK-MB, TC, TG, LDL-C and HDL-C in patients with AMI at the initial diagnosis. Subsequently, we performed co-expression network analysis to assess the relationship between these three lipid biomarkers and clinical indicators. The results suggested that there was a robust correlation between the three lipid biomarkers, while correlations with common clinical indicators were relatively weak (Figure 5D). Univariate and multivariate logistic regression analyses were utilized to detect the relationship between clinical index, lipid biomarkers and the risk of AMI. Univariate logistic regression analysis showed that CK-MB and three lipid biomarkers were risk factors for AMI in the discovery set (Table 2). Multivariate logistic regression analysis indicated that these three lipid biomarkers were independent risk factors for AMI (Table 2). Univariate and multivariate logistic regression analyses were performed in the validation set, which also showed that these three lipid markers were independent risk factors for AMI (Table 3). We analyzed the expression of these three lipid biomarkers in the discovery set and the validation set, respectively. The results showed that the three biomarkers were significantly up-regulated in patients with myocardial infarction in both the discovery set (Figure 7A) and the validation set (Figure 7B). We further followed up 60 AMI patients for 1 year, and 14 patients had a second hospital admission for any type of cardiovascular disorder. Subsequently, ROC analysis was performed to evaluate the efficacy of these three lipid biomarkers in predicting secondary admission in discharged patients with AMI. These three lipid biomarkers exhibited a strong predictive capacity in second admission, especially FFA (20:1) with an AUC of 0.662 (Figure 7C). It is worth noticing that the diagnostic model based on these three lipid biomarkers also showed a good predictive accuracy with an AUC of 0.685 (Figure 7D).

**Table 2:** Univariate and multivariate logistic regression in discovery set.

	**Univariate Analysis**		**Multivariate Analysis**	
	**OR (95% CI)**	***P*** **value**	**OR (95% CI)**	***P*** **value**
CK-MB	1.132 (1.040–1.233)	0.004	NA	NA
TC	0.970 (0.567–1.660)	0.913	NA	NA
TG	0.677 (0.404–1.137)	0.140	NA	NA
TC/TG	1.195 (0.944–1.513)	0.139	NA	NA
HDL-C	1.156 (0.448–2.979)	0.765	NA	NA
LDL-C	1.081 (0.589–1.986)	0.801	NA	NA
CarnitineC18:1-OH	2.394 (1.468–3.905)	<0.001	2.106 (1.212–3.659)	0.008
CarnitineC18:2-OH	7.502 (2.367–23.769)	<0.001	5.124 (1.415–18.557)	0.013
FFA (20:1)	2.682 (1.614–4.456)	<0.001	2.163 (1.231–3.800)	0.007

**Table 3:** Univariate and multivariate logistic regression in validation set.

	**Univariate Analysis**		**Multivariate Analysis**	
	**OR (95% CI)**	***P*** **value**	**OR (95% CI)**	***P*** **value**
CK-MB	1.145 (1.049–1.249)	0.002	1.156 (1.034–1.292)	0.011
TC	1.546 (0.959–2.490)	0.074	NA	NA
TG	1.107 (0.632–1.939)	0.723	NA	NA
TC/TG	1.298 (0.980–1.718)	0.069	NA	NA
HDL-C	0.677 (0.064–7.146)	0.746	NA	NA
LDL-C	1.724 (0.951–3.128)	0.073	NA	NA
CarnitineC18:1-OH	1.838 (1.291–2.615)	<0.001	1.960 (1.084–3.546)	0.026
CarnitineC18:2-OH	2.927 (1.459–5.870)	0.002	4.839 (1.127–20.774)	0.034
FFA (20:1)	2.157 (1.455–3.196)	<0.001	2.098 (1.274–3.454)	0.004

## Discussion

AMI is the most common cardiovascular disease with high mortality and disability world-wide, and its incidence presents a steady increase in developed and developing countries (30). The impact of AMI on global health is enormous with an estimated incidence of 65/100000, and ~8 million deaths attributed to AMI each year (https://www.who.int/). Over the past decade, the incidence of AMI has reached 60/100000 in China, and more than 3 million people die of AMI every year (31, 32). Without prompt treatment, the patients suffering from AMI may die within 10 min to several hours, and further there is 30% mortality during hospitalization (33). Moreover, the prognosis of AMI is not favorite, and the occurrence of AMI can increase the risk of heart failure (34). Therefore, early warning is very important to improve the prognosis of AMI.

In this study, 212 differentially expressed lipid metabolites (mainly glycerol phospholipids, acyl carnitine, and lysophosphatides) were identified via lipid metabolomics between the AMI group and the HC group. Many studies have shown that these lipid molecules were related to angiogenesis, inflammatory response and activation of apoptosis signals. KEGG analysis showed that the 212 differentially expressed lipid metabolites were mainly enriched in metabolic pathways, glycerophospholipid metabolism, glycolipid metabolism, and fat digestion and absorption that have been reported to be related to coronary artery disease. In addition, 58 differential lipid metabolites were identified from the comparison between AMI group and the UA group, of which 25 differential lipid metabolites were common between the AMI group and the HC group, and mainly included CAR, FFA, PC and TG. KEGG enrichment analysis showed that the 25 common differential lipid metabolites were associated with fatty acids, cholesterol and energy metabolism that have been demonstrated to play crucial role in the occurrence of coronary artery disease and AMI. Taken together, these results demonstrated significant differences in the lipid metabolic profile among AMI patients, UA patients and HC subjects, which may provide clues for further understanding the role of lipids in the occurrence and progression of AMI.

Zhong et al. evaluated the diagnostic efficacy of four differential metabolites, including trimer amine, choline, creatinine and carnitine in early AMI via LC-MS/MS targeting technology (35). In 2019, Goulart et al. identified 41 differential metabolites between 19 HC subjects and 15 patients with AMI, and then explored their potential function through bioinformatics analysis (36). Zhu et al. screened 10 abnormally expressed metabolites in myocardial infarction through LC-MS/MS, however, the diagnostic power of these 10 metabolites was limited due to the lack of validation (37). In 2020, Ali et al. identified differential metabolites between 20 AMI patients and 15 HC subjects by time-of-flight mass spectrometry (TOF-MS), and explored their potential metabolic function (38). Even though these studies have identified the differential metabolites related to AMI to some extent, these studies were performed by qualitative or semi quantitative analysis, and the diagnostic efficacy of the potentially identified metabolic biomarkers is not sufficient. Lipid metabolites have become promising potential biomarkers for several diseases. In the present study, we employed a highly sensitive mass spectrometer to quantitatively detect lipid metabolites. Stable isotope labeled and internal standard were used for the absolute quantitative detection of metabolites, which improves the accuracy, credibility and repeatability of the detection results. LAASO regression and SVM are commonly used in clinical research to identify characteristic variables, while their working principles are different. In this study, SVM was used to reduce the set of predictors for LASSO regression training in order to obtain more persuasive and robust biomarkers. Finally, three potential lipid biomarkers [Carnitine C18:1-OH, Carnitine C18:2-OH, FFA (20:1)] were selected from the 25 differential metabolites. These three lipid biomarkers and their combined diagnostic model presented robust performance in distinguishing AMI patients from UA patients with an AUC of about 0.9 in the discovery set. As expected, the three lipid biomarkers demonstrated well discrimination between AMI patients and UA patients in the validation and the whole set. Univariate and multivariate logistic regression analyses suggested that the three lipid biomarkers could provide diagnostic information independent of known clinical risk factors, including TC, TG, HDL-C, LDL-C, and CK-MB. Furthermore, these three lipid biomarkers to a certain extent have demonstrated prognostic efficacy in the second hospitalization attributed to cardiovascular disease within 1 year, and the effective rate was higher than 0.6. In the present study, we identified three novel potential lipid biomarkers for the clinical diagnosis of AMI.

Among the three potential lipid biomarkers, Carnitine C18:1-OH and Carnitine C18:2-OH belong to long-chain acyl carnitine, which is the intermediate of fatty acid metabolism in the mitochondria and is responsible for transporting fatty acyl to the mitochondria for energy metabolism (39). Bing et al. reported that 8 acyl carnitines significantly increased in schizophrenia patients and had a potential correlation with multiple metabolic pathways (40). Deung-Dae et al. showed that the heart function of Zebrafish treated with long-chain acyl carnitine was seriously damaged, and its mitochondrial function and ATP production were significantly affected (41). Sahir et al. showed that long-chain acyl carnitine accumulated in patients with end-stage renal disease, and aberrant expression of long-chain acyl carnitine could predict death due to cardiovascular disease (42). Increased circulating levels of long-chain acyl carnitine have been demonstrated to be linked to the occurrence of cardiovascular disease (43). In addition, long-chain acyl carnitine could elevate calcium outflow from isolated cardiac sarcoplasmic reticulum vesicles in a concentration dependent manner (44). Our results also suggested that CarnitineC18:1-OH and CarnitineC18:2-OH (two long-chain acyl carnitines) were significantly accumulated in AMI patients, compared with HC subjects and UA patients, and their abnormal expression was an independent risk factor for AMI. Although the specific mechanism of elevated long-chain acyl carnitine in cardiovascular diseases is largely unclear, it may be speculated that the reduction of long-chain acyl carnitine in daily diet or drugs could be a realistic and practical intervention to decrease the occurrence of AMI and other cardiovascular diseases.

Serum levels of FFA are mainly metabolized by triacylglycerol, which can provide energy for the body (45). Plasma FFA could be bound to albumin, and the elevated serum FFA level can increase the risk of cardiovascular disease (46). Recent studies have shown that excessive FFA can cause plasma membrane damage, which in turn induces myocardial dysfunction (47). However, not all excessive FFA is related to the occurrence of cardiovascular diseases (48). Medium and short chain fatty acids mainly provide energy to the body through oxidative metabolism, and long chain fatty acids are more vulnerable to cause oxidative damage due to their different chemical structures. It has been reported that an increase of plasma long-chain FFA concentration is not only a risk factor of cardiovascular disease, but also an independent risk factor of cardiovascular death (40). In this study, we found significantly higher plasma FFA (20:1) concentration in AMI patients, compared to HC subjects and UA patients, and ROC analysis showed that it has an excellent performance in distinguishing AMI patients. Of note, FFA (20:1) also had a prominent performance in predicting the second hospitalization of patients with AMI due to cardiovascular disease within 1 year, with an AUC of 0.662. These results suggested that FFA (20:1) can play an important role in predicting the occurrence of AMI, and can act as a promising novel AMI risk index.

Nevertheless, there are still some limitations to be addressed. First, the numbers of patients included in the percent study was limited. Therefore, our findings need to be further verified in a larger prospective cohort. Second, these biomarkers were detected under laboratory conditions, of which the quantification, calibration and robustness still needs to be further evaluate. Third, mass spectrometry analyses were performed using a SCIEX Triple Quad 6500+ LC-MS/MS, which has not been clinically validated. Therefore, in order to translate these results into clinical application, a fast and reproducible method should be explored.

Taken together, this study demonstrated significant differences in the plasma metabolic profile among AMI patients, UA patients, and HC subjects. CarnitineC18:1-OH, CarnitineC18:2-OH and FFA (20:1) were identified as new potential biomarkers for AMI, which can be used as early warning and prognosis evaluation of AMI.

## Data Availability Statement

The original contributions presented in the study are included in the article/supplementary material, further inquiries can be directed to the corresponding author/s.

## Ethics Statement

The studies involving human participants were reviewed and approved by Yue Bei People's Hospital. The patients/participants provided their written informed consent to participate in this study.

## Author Contributions

J-CL, JL, LT, and SZ conceived the study and drafted the manuscript. JL, QL, SZ, YY, W-JD, Q-GX, and Y-XC all participated in sample collection and data processing. J-CL, JL, and LT proofread and polished the manuscript. All authors read and approved the final manuscript. All authors contributed to subsequent drafts and approved the submitted version.

## Conflict of Interest

The authors declare that the research was conducted in the absence of any commercial or financial relationships that could be construed as a potential conflict of interest.

## Publisher's Note

All claims expressed in this article are solely those of the authors and do not necessarily represent those of their affiliated organizations, or those of the publisher, the editors and the reviewers. Any product that may be evaluated in this article, or claim that may be made by its manufacturer, is not guaranteed or endorsed by the publisher.
